# Supplementary material for: Flavonoid and Phenolic Quantification from Açaí (Euterpe oleracea Mart and Euterpe precatoria Mart), Mirití (Mauritia flexuosa L.), and Cupuassu (Theobroma grandiflorum (Wild. Ex Spreng.) Schum) from Vaupés, Colombia, Using LC-QqQ-MS
Source: Plants (Basel). 2025 Aug 24;14(17):2632. doi: 10.3390/plants14172632 (PMC12429868; doi:10.3390/plants14172632)

Table S1. Commercial standards (Phenolic acids and Flavonoids MetaSci®

Chemical Name	CAS #	Purity	Exact Mass	Molecular formula
**Phenolic acids and alcohols**				
2,3-Dihydroxybenzoic acid	303-38-8	98%	1.540.266	C7H6O4
Chlorogenic acid	327-97-9	≥95%	3.540.951	C16H18O9
Syringic acid	530-57-4	98%	1.980.528	C9H10O5
p-Coumaric acid	7400-08-0	99.8%	1.640.473	C9H8O3
m-Hydrocoumaric acid	621-54-5	95.1%	166.063	C9H10O3
Ferulic acid	1135-24-6	99.9%	1.940.579	C10H10O4
Sinapic acid	530-59-6	98%	2.240.685	C11H12O5
Resorcinol	108-46-3	>99.9%	1.100.368	C6H6O2
4-Hydroxybenzoic acid	99-96-7	98.5%	1.380.317	C7H6O3
Chemical Name	CAS #	Purity	Exact Mass	Molecular formula
2,6-Dihydroxybenzoic acid	303-07-1	>99.9%	1.540.266	C7H6O4
Dihydrocaffeic acid	1078-61-1	>99.9%	1.820.579	C9H10O4
Caffeic acid	331-39-5	>99.9%	1.800.423	C9H8O4
Phloretic acid	501-97-3	>99%	166.063	C9H10O3
Hydroferulic acid	1135-23-5	99.3%	1.960.736	C10H12O4
Ellagic acid dihydrate	476-66-4	>99%	3.020.063	C14H6O8
5-Methoxysalicylic acid	4/02/2612	98%	1.680.423	C8H8O4
Gentisic acid	490-79-9	>99%	1.540.266	C7H6O4
4-Acetocatechol	1197-09-7	>99.9%	1.520.473	C8H8O3

4-Methylcatechol	452-86-8	>99.9%	1.240.524	C7H8O2
Acetylphloroglucinol	480-66-0	>99.9%	1.680.423	C8H8O4
Salicylic acid	69-72-7	98%	1.380.317	C7H6O3
trans-2-Hydroxycinnamic acid	614-60-8	>99%	1.640.473	C9H8O3
Caffeic acid dimethyl ether	2316-26-9	98%	2.080.736	C11H12O4
Gallic acid	149-91-7	99.5%	1.700.215	C7H6O5
3,5-Dihydroxybenzoic acid	99-10-5	>99.9%	1.540.266	C7H6O4
Vanillic acid	121-34-6	99.6%	1.680.423	C8H8O4
Nordihydroguaiaretic Acid	500-38-9	95%	3.021.518	C18H22O4
Terephthalic acid	100-21-0	>99%	1.660.266	C8H6O4
4-Acetylresorcinol	89-84-9	>99.9%	1.520.473	C8H8O3
Rosmarinic acid	20283-92-5	>99.9%	3.600.845	C18H16O8
Caffeic acid phenethyl ester	104594-70-9	98%	2.841.049	C17H16O4
2,3,4-Trihydroxybenzoic acid	610-02-6	84.2%	1.700.215	C7H6O5
2,4-Dihydroxybenzoic Acid	89-86-1	>99.9%	1.540.266	C7H6O4
3-Hydroxybenzoic acid	99-06-9	>99.9%	1.380.317	C7H6O3
m-Coumaric acid	14755-02-3	>99.9%	1.640.473	C9H8O3
2-Acetylresorcinol	699-83-2	>99.9%	1.520.473	C8H8O3
3,4,5-Trimethoxycinnamic acid	90-50-6	>99.9%	2.380.841	C12H14O5

Flavonoids				
(+)-Catechin (Hydrate)	154-23-4	0.9696	290.079	C15H14O6
Mangiferin	4773-96-0	0.95689	4.220.849	C19H18O11
Naringin	10236-47-2	0.98792	5.801.792	C27H32O14
Myricetin	529-44-2	0.97556	3.180.376	C15H10O8
Baicalin	21967-41-9	0.92862	4.460.849	C21H18O11
Quercetin	117-39-5	0.972	3.020.427	C15H10O7
Neohesperidin dihydrochalcone	20702-77-6	0.973	6.122.054	C28H36O15
Kaempferol	520-18-3	0.988	2.860.477	C15H10O6
Baicalein	491-67-8	0.991	2.700.528	C15H10O5
(-)-Epigallocatechin gallate hydrate	989-51-5	0.994	4.580.849	C22H18O11
Chemical Name	CAS #	Purity	Exact Mass	Molecular formula
Polydatin	65914-17-2	0.996	3.901.315	C20H22O8
Hesperidin	520-26-3	0.97	6.101.898	C28H34O15
Diosmin	520-27-4	0.978	6.081.741	C28H32O15
Morin	480-16-0	0.962	3.020.427	C15H10O7
(+/-)-Naringenin	67604-48-2	0.979	2.720.685	C15H12O5
Luteolin	491-70-3	0.994	2.860.477	C15H10O6
Hesperetin	520-33-2	0.987	302.079	C16H14O6
Diosmetin	520-34-3	>99.9%	3.000.634	C16H12O6
Chrysin	480-40-0	>99.9%	2.540.579	C15H10O4
(-)-Epicatechin	490-46-0	0.958	290.079	C15H14O6
Phloridzin	60-81-1	0.995	436.137	C21H24O10
Fisetin	528-48-3	0.928	2.860.477	C15H10O6
Daidzein	486-66-8	0.977	2.540.579	C15H10O4
Resveratrol	501-36-0	0.99	2.280.786	C14H12O3
Apigenin	520-36-5	0.997	2.700.528	C15H10O5
(+)-Taxifolin	480-18-2	0.978	3.040.583	C15H12O7
Puerarin	3681-99-0	>99.9%	4.161.107	C21H20O9
Rutin	153-18-4	0.982	6.101.534	C27H30O16
Neohesperidin	13241-33-3	0.991	6.101.898	C28H34O15
Naringin dihydrochalcone	18916-17-1	>99.9%	5.821.949	C27H34O14

Equol	94105-90-5	0.935	2.420.943	C15H14O3
Genistein	446-72-0	>99.9%	2.700.528	C15H10O5
Isoliquiritigenin	961-29-5	0.911	2.560.736	C15H12O4
Formononetin	485-72-3	0.989	2.680.736	C16H12O4
Biochanin A	491-80-5	>99.9%	2.840.685	C16H12O5

Table S2. Collision energy and transitions used in multiple reaction monitoring (MRM) for the determination of phenolic compounds

**Compound**	**Transition (m/z)**	**tR (min)**	**Fragmentor**	**CE (V)**
Gallic acid	169.0 -> 125.0	1.07	100	20
Mangiferin	420.9 -> 301.0	1.21	100	22
Puerarin	415.0 -> 267.0	1.23	100	38
3,5-Dihydroxybenzoic acid	152.9 -> 108.9	1.23	100	10
Chlorogenic acid	353.0 -> 191.0	1.24	100	30
2,3,4-Trihydroxybenzoic acid	168.9 -> 150.9	1.32	100	10
(+)-Catechin (Hydrate)	289.0 -> 245.0	1.35	100	10
(-)-Epicatechin	289.0 -> 109.0	1.54	100	30
(-)-Epigallocatechin gallate hydrate	457.0 -> 169.1	1.57	100	20
Dihydrocaffeic acid	180.9 -> 136.9	1.59	100	10
4-Hydroxybenzoic acid	137.0 -> 93.0	1.67	100	10
Phloretic acid	165.0 -> 121.0	1.70	80	10
Terephthalic acid	165.0 -> 121.0	1.70	100	10
Caffeic acid	179.0 -> 135.0	1.71	100	10
Syringic acid	197.0 -> 182.0	1.76	80	15
Gentisic acid	153.0 -> 108.0	1.78	100	20
4-Acetocatechol	151.0 -> 108.0	1.79	100	20
Vanillic acid	167.0 -> 151.9	1.79	80	20
Resorcinol	109.0 -> 65.0	1.94	100	10
2,6-Dihydroxybenzoic acid	153.0 -> 109.0	1.95	100	10
Rutin	609.0 -> 300.1	2.02	100	42
3-Hydroxybenzoic acid	137.0 -> 93.0	2.03	100	10
2,4-Dihydroxybenzoic Acid	153.0 -> 109.0	2.03	100	10
2,3-Dihydroxybenzoic acid	153.0 -> 109.0	2.05	100	20
Ellagic acid dihydrate	301.0 -> 145.1	2.38	100	40
Polydatin	389.0 -> 227.0	2.46	100	10
p-Coumaric acid	163.0 -> 119.0	2.55	100	20
Hydroferulic acid	195.0 -> 136.0	2.64	100	20
Sinapic acid	223.0 -> 193.0	2.83	100	30
m-Hydrocoumaric acid	165.0 -> 121.0	2.93	100	10
Ferulic acid	193.0 -> 134.0	2.97	100	20
(+)-Taxifolin	303.0 -> 285.0	3.27	100	10
4-Methylcatechol	123.0 -> 108.0	3.28	100	20
m-Coumaric acid	163.0 -> 119.0	3.51	100	10
Naringin	579.0 -> 271.0	3.87	100	34
Acetylphloroglucinol	167.0 -> 123.0	3.96	100	20

Diosmin	607.0 -> 299.1	4.05	100	26
Hesperidin	609.0 -> 301.0	4.41	100	26
4-Acetylresorcinol	151.0 -> 91.0	4.80	100	20
trans-2-Hydroxycinnamic acid	163.0 -> 119.0	4.90	100	10
Neohesperidin	609.1 -> 286.1	5.29	100	50
Rosmarinic acid	359.0 -> 161.0	5.31	100	20
Myricetin	316.9 -> 151.0	5.38	100	26
Fisetin	285.0 -> 135.0	5.47	100	20
Salicylic acid	137.0 -> 93.0	5.62	100	20
5-Methoxysalicylic acid	167.0 -> 107.9	6.00	100	20
Phloridzin	435.0 -> 273.0	6.07	100	14
Caffeic acid dimethyl ether	207.0 -> 103.0	6.97	80	10
Resveratrol	226.9 -> 185.0	7.11	100	20
2-Acetylresorcinol	151.0 -> 91.0	7.79	100	20
Baicalin	444.9 -> 269.0	8.07	100	18
Naringin dihydrochalcone	581.0 -> 273.0	8.28	100	40
Daidzein	252.9 -> 224.0	8.74	100	40
3,4,5-Trimethoxycinnamic acid	237.0 -> 102.9	9.54	100	20
Neohesperidin dihydrochalcone	611.0 -> 303.0	9.93	100	38
Luteolin	284.9 -> 133.0	10.21	100	38
Morin	301.0 -> 121.0	10.27	100	26
Quercetin	300.9 -> 121.0	10.27	100	30
(+/-)-Naringenin	270.9 -> 150.9	11.45	100	42
Genistein	268.9 -> 133.1	11.46	100	34
Apigenin	268.9 -> 151.0	11.52	100	26
Equol	241.0 -> 118.9	11.52	100	20
Phloretin	273.0 -> 167.0	11.56	100	14
Kaempferol	284.9 -> 93.1	11.74	100	38
Diosmetin	299.0 -> 284.1	11.80	100	18
Hesperetin	301.0 -> 163.9	11.89	100	26
Baicalein	269.0 -> 137.0	12.13	100	50
Isoliquiritigenin	255.0 -> 119.0	12.46	100	26
Formononetin	267.0 -> 252.0	12.68	100	34
Nordihydroguaiaretic Acid	301.0 -> 122.0	13.25	100	30
Chrysin	252.9 -> 63.1	13.75	100	38
Caffeic acid phenethyl ester	283.0 -> 135.0	14.13	135	20
Biochanin A	282.9 -> 268.0	14.17	100	20

Figure S1. Chromatograms of phenolic and flavonoid compounds obtained by liquid chromatography for two acai species

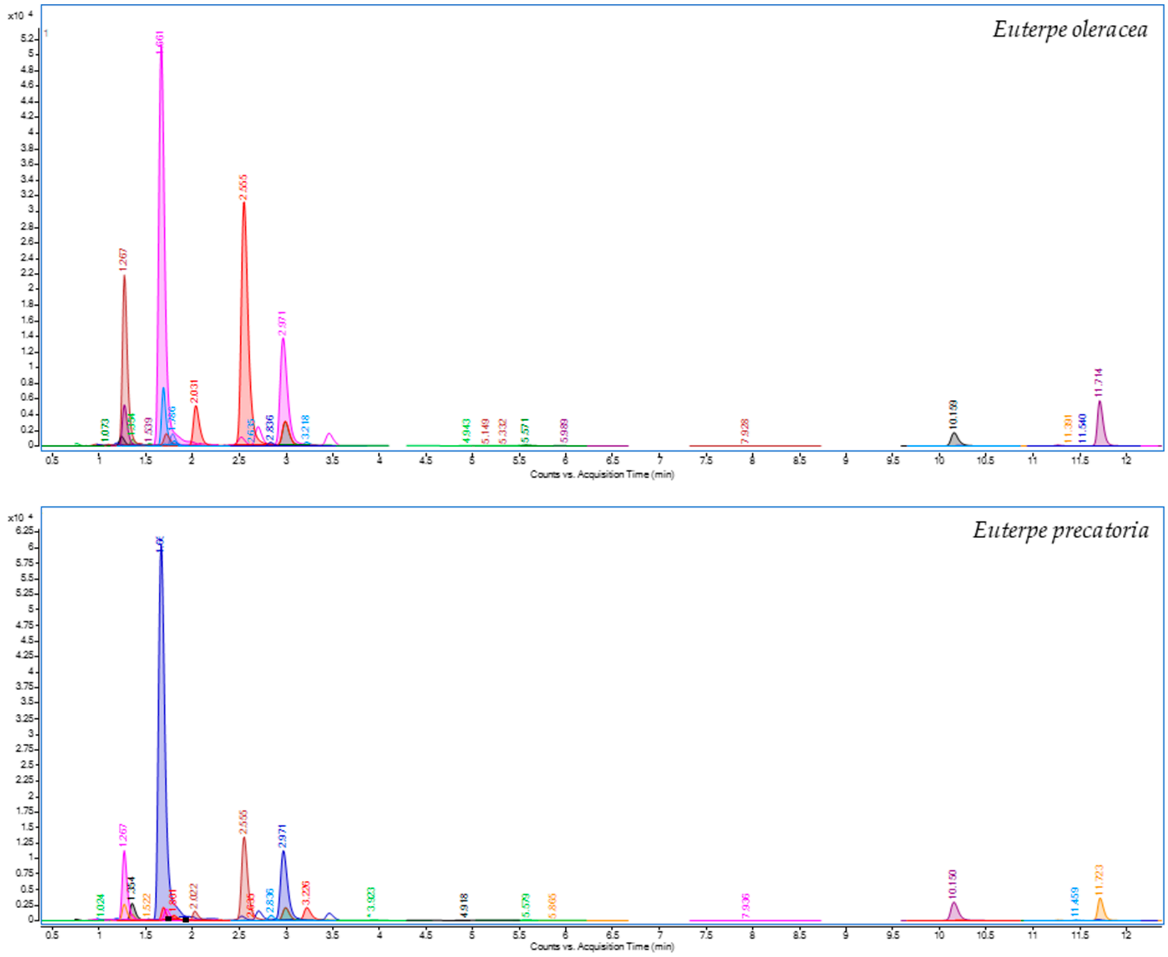

Figure S2. Chromatograms of phenolic and flavonoid compounds obtained by liquid chromatography for cupuassu and mirití

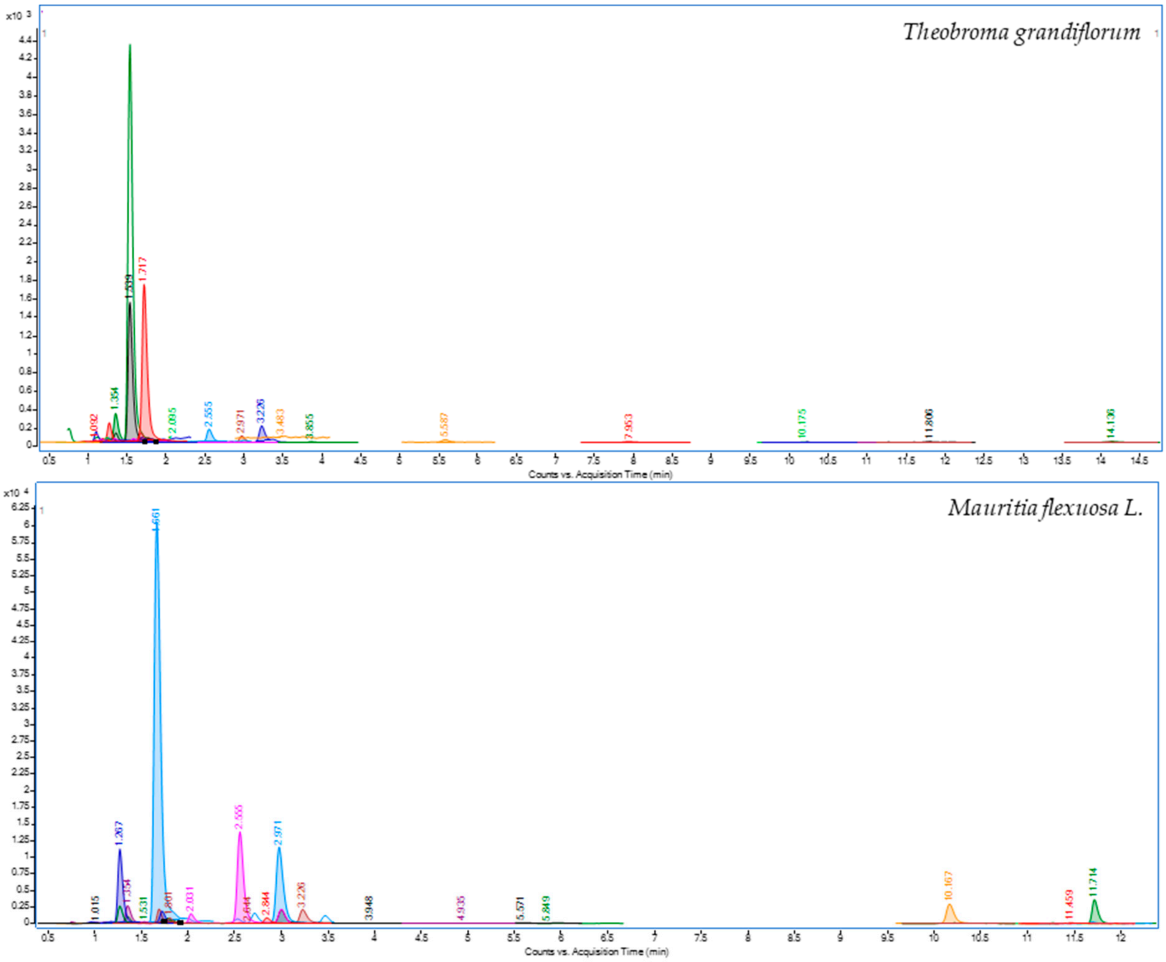

Supplement: Supplementary file 1 [file plants-14-02632-s001.zip › plants-3796706-supplementary.pdf]
